# De-obstruction of bladder outlet in humans reverses organ remodelling by normalizing the expression of key transcription factors

**DOI:** 10.1186/s12894-024-01417-8

**Published:** 2024-02-07

**Authors:** Akshay Akshay, Ali Hashemi Gheinani, Mustafa Besic, Sophie Braga, Anne-Christine Uldry, Manfred Heller, Hubert Rehrauer, Catharine Aquino Fournier, Fiona C. Burkhard, Katia Monastyrskaya

**Affiliations:** 1 Functional Urology Research Laboratory, Department for BioMedical Research DBMR, University of Bern, Bern, Switzerland; 2 Graduate School for Cellular and Biomedical Sciences, University of Bern, Bern, Switzerland; 3 Department of Urology, Inselspital University Hospital, 3010 Bern, Switzerland; 4 Department of Urology, Boston Children’s Hospital, Department of Surgery, Harvard Medical School, Boston, MA USA; 5 Broad Institute of MIT and Harvard, Cambridge, MA USA; 6 Proteomics and Mass Spectrometry Core Facility, DBMR University of Bern, Bern, Switzerland; 7 Functional Genomics Center Zurich, ETH Zurich and University of Zurich, Zurich, Switzerland

**Keywords:** Bladder, Obstruction, Gene expression, Urodynamics, Omics

## Abstract

**Background:**

Benign prostatic hyperplasia in elderly males often causes bladder outlet obstruction termed benign prostatic obstruction (BPO). BPO induces lower urinary tract symptoms and quantifiable urodynamic alterations in bladder function. When conservative medical treatments are exhausted, surgical interventions like transurethral resection of the prostate (TURP) are employed for bladder outlet de-obstruction. Elucidating the molecular changes in the human bladder resulting from BPO and their reversal post-de-obstruction is pivotal for defining the “point of no return”, when the organ deterioration becomes irreversible. In this study we carried out a comprehensive molecular and urodynamic characterization of the bladders in men with BPO before TURP and 3 months after the relief of obstruction.

**Methods:**

We report integrated transcriptome and proteome analysis of bladder samples from male patients with BPO before and 3 months after de-obstruction surgery (TURP). mRNA and protein profiles were correlated with urodynamic findings, specifically voiding detrusor pressure (PdetQmax) before TURP. We delineated the molecular classifiers of each group, pointing at the different pre-TURP bladder status.

**Results:**

Age-matched patients with BPO without DO were divided into two groups based on the PdetQmax values recorded by UDI before de-obstruction: high and medium pressure (HP and MP) groups. Three months after de-obstruction surgery, the voiding parameters PdetQmax, Qmax and RV were significantly improved in both groups, without notable inter-group differences in the values after TURP. Patients with high PdetQmax showed less advanced remodeling and inflammatory changes than those with lower values. We detected significant dysregulation of gene expression, which was at least partially reversed by de-obstruction in both patients’ groups. Transcription factor SOX21 and its target thrombospondin 4 (THBS4) demonstrated normalization post-TURP.

**Conclusions:**

Our findings reveal substantial yet incomplete reversal of cell signalling pathways three months after TURP, consistent with improved urodynamic parameters. We propose a set of biomarker genes, indicative of BPO, and possibly contributing to the bladder changes. This study unveils the stages of progressive obstruction-induced bladder decompensation and offers insights into selecting an optimal intervention point to mitigate loss of contractility.

**Supplementary Information:**

The online version contains supplementary material available at 10.1186/s12894-024-01417-8.

## Background

Benign prostatic hyperplasia can, independent of prostate size, cause bladder outlet obstruction (BOO) in elderly males termed benign prostatic obstruction (BPO). BPO is a dynamic chronic process accompanied by lower urinary tract symptoms (LUTS), including storage symptoms such as frequency, urgency and nocturia and voiding symptoms such as weak stream, delayed or intermittent voiding and incomplete emptying. Moderate to severe LUTS are found in approximately 25% of men aged 40 to 49 and in 50% aged 70 to 79 [1]. After exhausting less invasive medical treatment options, patients are offered surgical treatment, for example transurethral resection of the prostate (TURP) to de-obstruct the bladder outlet. Although voiding parameters significantly improve, 20 to 40% of patients continue to experience at least some bothersome LUTS [2, 3], inciting further research into the factors contributing to BPO-induced bladder dysfunction. Specifically, identifying the men at risk of irreparable bladder damage due to BPO and optimal timing of de-obstruction surgery are paramount to avoid loss of bladder function. Limited evidence from human studies and animal models, summarized in a recent report [4], supports the notion that BPO gradually progresses from inflammation to hypertrophy to fibrosis [5]. BPO-induced bladder remodeling includes initial bladder hypertrophy during the compensated stage characterized by the increased detrusor contractility / pressure during voiding, and can be accompanied by detrusor overactivity (DO). This ultimately can lead to loss of bladder function (detrusor underactivity) [4].

In humans, the advancement of BPO-induced bladder remodelling is impossible to monitor, because several years can pass between the onset of symptoms and presentation in clinic. Nevertheless, it is possible to establish a correlation between the urodynamic phenotypes of BPO-induced lower urinary tract dysfunction (LUTD) and the molecular alterations in the bladder, as we have recently shown in a comprehensive study of human bladder biopsies, obtained before TURP [6]. The overall number of gene expression changes increased progressively: It was the lowest in BPO with detrusor overactivity and the highest in underactive decompensated bladders [6] compared to controls without LUTD. Animal models of LUTD cannot replicate the chronic gradual longitudinal changes seen in human disease. To mimic human bladder outlet obstruction in rodents, the urethra is loosely ligated creating a partial bladder outlet obstruction (pBOO). In contrast to humans this causes acute obstruction resulting in a significant initial inflammatory impact immediately after surgery. There are other species-specific differences in immune processes, for example, the TNF-alpha-induced changes in human BPO are not observed in mouse pBOO, indicating a different pathophysiological mechanism of organ remodelling [7]. Similarly, the acute partial obstruction induced in animals results in bladder stretch which is rarely encountered in humans and may affect the results generated by this model.

Alternatively, it is possible to monitor both the functional and molecular changes in the bladder after surgical de-obstruction, and this was done in a number of animal studies, mostly in larger rodents such as rats [8, 9], guinea pigs [10] and rabbits [11]. It was noted that despite the overall improvement of the micturition parameters, complete restoration of bladder function did not occur [12], and many molecular changes persisted after the relief of obstruction in animals [13]. Similarly, in a large proportion of human patients, followed after TURP, removal of obstruction improved symptom scores and flow rate [14], but did not completely reverse the LUTD evident by persistent DO [15] and low or inadequate detrusor contractility [16].

Understanding the molecular alterations in the human bladder caused by BPO and persisting after the relief of obstruction is indispensable for defining the “point of no return”, when the organ deterioration becomes irreversible. This should help identify new therapeutic options, including correct timing of de-obstruction surgery. As a follow-up of our earlier study, which revealed molecular networks, hubs of signalling, and biomarkers in BPO-induced bladder dysfunction in men with defined functional phenotypes [6], we now report a comprehensive molecular and urodynamic characterization of the bladders in men with BPO before TURP and 3 months after the relief of obstruction. We performed an integrated transcriptome and proteome analysis of the bladder biopsies in the two patient groups with a significant difference in the voiding detrusor pressure (PdetQmax), and delineated the molecular classifiers of each group, pointing at the different pre-TURP bladder status. The gene expression follow-up 3 months after surgery sheds light on the processes, contributing to the recovery of bladder function and transcription factors, involved in the regulation of bladder remodelling in BPO.

## Methods

### Patient selection, biopsy collection and RNA isolation

Voiding symptoms were assessed in all patients by IPSS (International Prostate Symptom Score) and 48 hours urinary diary, urinary free flow and sonographic measurements of residual urine volumes [17]. In controls O’Leary-Sant symptom index, O’Leary-Sant problem index and pelvic pain VAS scores were additionally assessed.

#### Group 1 - normal bladder function, designated “control”

Controls were mostly recruited from patients undergoing cystoscopy during stone treatment and qualified with the following results: IPSS score < 8, O’Leary-Sant symptom index < 6, O’Leary-Sant problem index < 6, pelvic pain VAS score < 4, voiding frequency < 8/24 h assessed by bladder diary, a bell-shaped flow curve and no post-void residual urine (*n* = 6). Ethical approval was given for cold cup biopsies from patients with no history of LUTS undergoing an invasive procedure in anesthesia for stone treatment.

#### Groups 2 and 3, bladder outlet obstruction high pressure (HP) and medium pressure (MP) groups

In all patients with LUTS due to BOO urodynamic studies were performed according to the International Continence Society (ICS) standards. Bladder contractility and BOO were assessed simultaneously. Additional cystoscopy was performed to further assess the obstructive component of the prostate and to exclude bladder tumours. Groups were defined according to ICS terminology after all urodynamic results were reviewed by a second experienced functional urologist as follows:

#### Group 2 – BOO without DO with high PdetQmax, designated “HP”

Patients with increased detrusor pressure and reduced urine flow during pressure flow studies without involuntary detrusor contractions during the filling phase (phasic and/or terminal) and defined as obstructed according to the Abrams-Griffith nomogram and the BOOI. Patients with maximal detrusor pressure at maximal flow during voiding (PdetQmax) ≥ 90 cmH2O were included in this group (*n* = 3).

#### Group 3 – BOO without DO with medium PdetQmax, designated “MP”

Patients with increased detrusor pressure and reduced urine flow during pressure flow without involuntary detrusor contractions during the filling phase (phasic and/or terminal) and defined as obstructed according to the Abrams-Griffith nomogram and the BOOI. Patients with a detrusor pressure at maximal flow (PdetQmax) of < 90 cmH2O were included in this group (*n* = 3).

In all groups four urothelium covered muscle containing cold-cup biopsies were collected from the bladder dome by the same urologist. Biopsies were stored in RNAlater at − 70 °C until RNA or protein isolation. Three months after TURP, bladder function in the HP and MP groups was re-assessed by UDI, and a second set of bladder dome biopsies collected and stored in RNAlater.

### mRNA sequencing and alignment

Total RNA was isolated using mirVana kit (Applied Biosystems) as described previously [18]. RNA was treated with DNAse (DNA-free kit, Ambion), its quality controlled by BioAnalyzer and further processed for library preparation and NGS as described in our previous study [6]. Briefly, sequencing was performed on the Illumina HiSeq 2000 single end 100 bp using the TruSeq SBS Kit v3-HS (Illumina, Inc., California, USA). Read mapping to human reference genome hg38 was done using STAR (version 2.7.0e). Counting the number of reads/gene was done using featureCounts [19] library in R (version 3.6.1).

### Differential expression and transcription factor analysis

Differentially expressed genes were identified using the Bioconductor packages DESeq2 (version 1.30.1) [20] and edgeR (version 3.32.1) [21]. Genes with adjusted *p*-value < 0.1 were considered as significantly DEGs, The tftargets (version 1.3) library in R was used to access TRED, ITFP, TRRUST, and Marbach [22–25] databases containing predicted and known human TF targets.

### Protein sample preparation and liquid chromatography tandem mass spectrometry

For the protein isolation from the RNAlater-preserved samples, the biopsies were submerged in 200 μl M-PER Mammalian protein extraction reagent (Thermo Scientific) and disrupted on ice using TissueRuptor homogenizer (Qiagen). After removing the tissue debris by centrifugation, and estimating the protein concentration by BCA assay, 30 μg of protein extracts were loaded on 12% SDS-PAGE and separated for about 1 cm. After Coomassie staining and destaining, the lane was cut into five horizontal slices. Proteins were in-gel digested as described elsewhere [26]. The digests were analysed by liquid chromatography LC/MS-MS (Easy1000 nanoLC coupled to a QExactive classic mass spectrometer, ThermoFisher Scientific) with one injection of 5-μl digests. Peptides were trapped on a C18 PepMap100 precolumn (5 μm, 100 Å, 300 μm × 5 mm, ThermoFisher Scientific, Reinach, Switzerland) and separated by backflush on a C18 column (3 μm, 100 A°C, 75 μm × 15 cm, Nikkyo Technos, Tokyo, Japan) by applying a 40-min gradient of 5% acetonitrile to 40% in water, 0.1% formic acid with a flow rate of 300 nl/min. The Full Scan method was set with a mass range of 360–1400 m/z, a resolution at 70,000 with an automatic gain control (AGC) target of 1 × 10^6^, and a maximum ion injection time of 50 ms. A data-dependent method for the 10 most intense precursor ion fragmentations was applied with the following settings: dynamic exclusion time of 20 s, resolution of 17,500, AGC of 1 × 10^5^, maximum ion time of 110 ms, isolation mass window of 2 m/z, normalized collision energy of 27%, under fill ratio 1%, charge exclusion of unassigned and 1+ ions, and peptide match preferred, respectively.

LC-MS/MS data were processed with MaxQuant (version 1.6.14.0) using default orbitrap settings for peak detection, strict trypsin cleavage rule, allowing up to three missed cleavages, variable oxidation on methionine, acetylation of protein N-termini, and deamidation of asparagine and glutamine, with fixed carbamidomethylation of cysteines, respectively. Match between runs was used with a retention time window of 0.7 min. The fragment spectra were interpreted using the SwissProt protein sequence database, release 2021_04. Protein identifications were accepted only if at least two razor peptides were identified at a 1% false discovery rate (FDR) cut-off on peptide spectrum match, peptide and protein level. Potential contaminants and protein groups only identified by site were removed prior to further analysis. Missing label-free (LFQ) values were imputed in the following manner: if there were at most 1 non missing value in a group of replicates, then the missing values in this group of replicates were imputed by drawing random values from a Gaussian distribution of width 0.3x sample standard deviation centred at the sample distribution mean minus 2.5x sample standard deviation; any remaining missing values were imputed by the Maximum Likelihood Estimation (MLE) [27]. Differential expression was performed by moderated t-test [28] for protein groups counting at least 2 detections in at least 1 group of replicates. Adjusted *p*-values for multiple testing were calculated by the Benjamini-Hochberg method [29]. Significance curves were obtained as in [30], such that |log2 fold| > =1 and adjusted p-value <=0.05 (0.05 reached at asymptotically high fold changes). The imputation procedure was repeated 20x, and protein groups found consistently differentially expressed with respect to the significance curve through the imputation cycles were especially flagged.

### Sample clustering and pathway analysis

#### Functional enrichment analysis

Gene Ontology (GO) over-representation analysis (ORA) [31] methods were used to gain biological insight on the DEGs. We used clusterProfiler (version 3.18.1) package [32] in R to perform ORA on GO terms associated with DEGs or DEPs. A threshold of *p*-value less than 0.1 was used to define statistical significance. Pathway analysis for differentially expressed proteins (DEPs) was carried out using “Ingenuity canonical pathway” tool in IPA (IPA®, QIAGEN Redwood City). To determine whether the activity of canonical pathways, including functional end-points, is increased or decreased based on differentially expressed proteins in the datasets, the pathway activity (z-score) was calculated by IPA.

#### Upstream regulator analysis

The upstream pathway analysis module of Ingenuity Pathway Analysis (IPA) (Application Build 377,306 M dated 2016-03-26, Content Version 27,216,297 build ing_idris dated 2016-03-16) was used. Overlap *p*-values were calculated by IPA using Fisher’s exact test, based on the significance of the overlap between the known targets and experimentally identified set of regulated genes. The supplementary file named “session-info” contains a comprehensive list of the utilized packages during the analysis, accompanied by their respective version numbers and citations.

## Results

### Patient grouping after urodynamic assessment

Patients were recruited and examined as described in Methods. Control group or “C” were mostly patients undergoing treatment for stone disease with normal bladder function. In all patients with LUTD due to BPO urodynamic studies (UDI) were performed and bladder contractility and BPO were assessed simultaneously. Based on questionnaires and urodynamic examination, patients without detrusor overactivity were selected. This group demonstrated increased detrusor pressure Pdet and reduced urine flow Qmax without involuntary detrusor contractions during the filling phase. After UDI, four urothelium-covered muscle-containing biopsies were collected from the bladder dome of each patient by the same urologist and total RNA isolated as described previously [18], or the biopsy was processed for proteomic analysis as described in Methods. The first set of biopsies was designated “before”. Three months after surgical de-obstruction, bladder function was examined by UDI, and the second sets of biopsies collected, designated “after”.

**Fig. 1:**
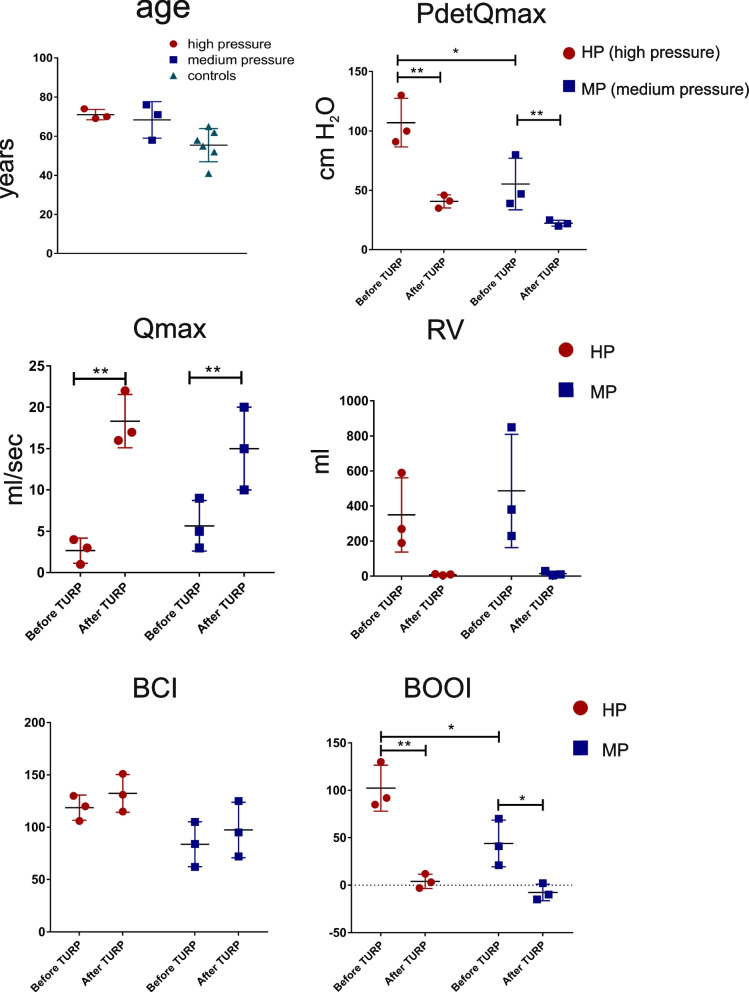
Age and urodynamic parameters in BPO patients before and after de-obstruction surgery. PdetQmax - maximal detrusor pressure at maximal flow during voiding (cm H_2_O), Qmax - maximal flow during voiding (ml/sec), RV - post-void residual urine volume. BCI - bladder contractility, calculated: BCI = Pdet Qmax + 5 Qmax. BOOI - the bladder outlet obstruction index, calculated: BOOI = Pdet @ Qmax − 2 Qmax. Statistical significance (*p < 0.05; **p < 0.01) is indicated

Men with BPO without DO were further sub-grouped based on the average detrusor pressure at maximum flow rate (PdetQmax). High pressure group (HP) (*n* = 3) had PdetQmax 107 ± 20.4 cmH2O before TURP, and PdetQmax 40.6 ± 5.5 cmH2O after TURP, while the medium pressure group (MP) (n = 3) had PdetQmax 55 ± 21.7 cmH2O before TURP, and PdetQmax 22 ± 2.64 cmH2O after TURP (Fig. 1). Both groups were similar in age (high pressure 71 ± 2.6 y.o., medium pressure 68 ± 9 y.o.), and in both groups TURP reduced the post-void residual urine volume to < 50 ml (RV) and improved the maximum flow rate (Qmax) (Fig. 1). Bladder contractility index (BCI) was calculated using formula BCI = PdetQmax + 5 Qmax [33]. BCI was normal and generally higher in HP group and weak in the MP group, and increased slightly in both groups after TURP, though the difference was not significant (Fig. 1). In contrast, the bladder outlet obstruction index (BOOI), represented by the equation: BOOI = PdetQmax − 2 Qmax [33], was significantly (*p* < 0.05) higher in HP group compared to the MP group before TURP, and TURP significantly (*p* < 0.001 and p < 0.05 in HP and MP groups, respectively) reduced BOOI to the values below 20, indicating the relief of obstruction (Fig. 1).

### Transcriptome analysis reveals differences between HP and MP groups

**Fig. 2:**
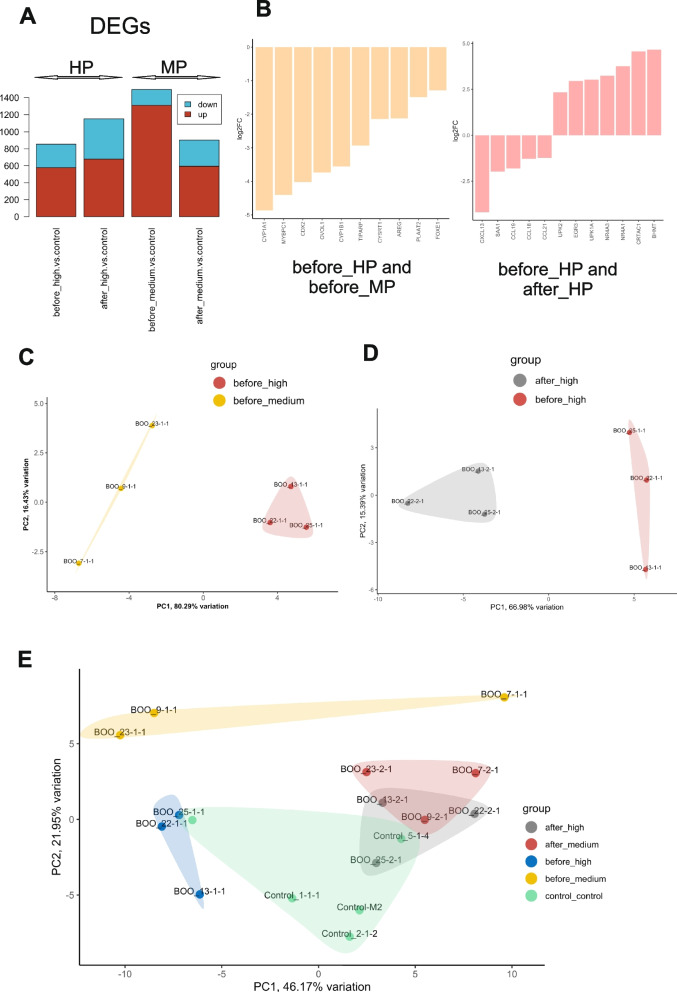
Differentially expressed mRNAs in BPO patients’ groups before and after TURP. **A** Total number of differentially expressed genes (DEGs) in HP and MP groups compared to controls (edgeR (adjusted *p*-value< 0.1)). **B** Group classifier genes, determined as an overlap between edgeR, DESeq2 and PCA as described in the text. The pair-wise comparisons HP vs. MP before TURP and HP before and after TURP are indicated. **C** Principal component analysis (PCA) based on normalized read counts of HP vs. MP group 10 gene classifiers. Before_high samples are shown as red labelled dots, and before_medium as yellow labelled dots. **D** PCA based on normalized read counts of HP before TURP vs HP after TURP 12 gene classifiers. Before_high samples are shown as red labelled dots, and after_high as grey labelled dots. **E** PCA applied to all available datasets including controls using the combination of both classifiers (22 genes)

Next-generation sequencing (NGS) was used to analyse the transcriptomes of bladder dome biopsies collected before and after TURP. Differentially expressed genes (DEGs) were determined in high and medium pressure groups compared to controls using DESeq2 (adjusted *p*-value< 0.1) and edgeR (adjusted p-value< 0.1). As per edgeR results, HP group, compared to controls, had on average 578 up- and 277 – down-regulated genes before TURP, and 679 up- and 472 – down-regulated genes after TURP, with the expression levels of 510 genes normalised (returned to control level) after de-obstruction, and 806 genes became de-novo regulated after TURP. MP group, compared to controls, had on average more DEGs before TURP (1310 up- and 186 down-regulated), with the overall number of DEGs diminishing after de-obstruction (1093 genes normalized and 500 became de-novo regulated) (Fig. 2A).

One of the objectives of this study was to select a robust set of genes that can effectively distinguish between HP and MP groups. To ensure the accuracy of our findings and minimize false positives, we employed two widely used tools, DESeq2 and edgeR, to identify DEGs between the groups. This approach enabled us to identify genes with significant differential expression in a highly confident manner. To further enhance the reliability of our results, we utilized Principal Component Analysis (PCA). By transforming the original features (genes) into a new set of variables known as principal components (PCs), PCA effectively captures the underlying patterns in the data. Using this approach, we selected the genes that fell within the top or bottom 20% of the loading range for the top six principal components (PCs). These selected genes are considered as most significant contributors to distinguish the studied groups. In the final stage of the analysis, we compared the genes identified as highly influential by PCA and the list of differentially expressed genes (DEGs) obtained using both DESeq2 and edgeR tools. The common DEGs intersection represented a set of genes that should serve as reliable classifiers for the investigated groups.

This pipeline yielded 10 genes discriminating between HP and MP groups before TURP (Fig. 2B, all genes expressed lower in HP dataset than in the MP dataset) and 12 genes, discriminating between “before” and “after”-TURP samples of HP group (7 were up- and 5 down-regulated in “after” HP group compared to the “before” HP group) (Fig. 2B). The PCA carried out using 10 HP classifier genes could reliably discriminate between HP and MP groups before TURP (Fig. 2C), while 12 de-obstruction classifier genes could discriminate between after- and before-TURP samples of the HP group (Fig. 2D). Interestingly, the combination of both classifiers (22 genes) applied to all available datasets including controls demonstrated a clear separation of “before” HP and “before” MP groups from each other and from controls. The samples collected 3 months after de-obstruction were more similar to each other and grouped closer to controls, indicating at least partial restoration of gene expression changes after the relief of obstruction (Fig. 2E).

### Effects of de-obstruction on dysregulated biological processes in the high pressure (HP) group

**Fig. 3:**
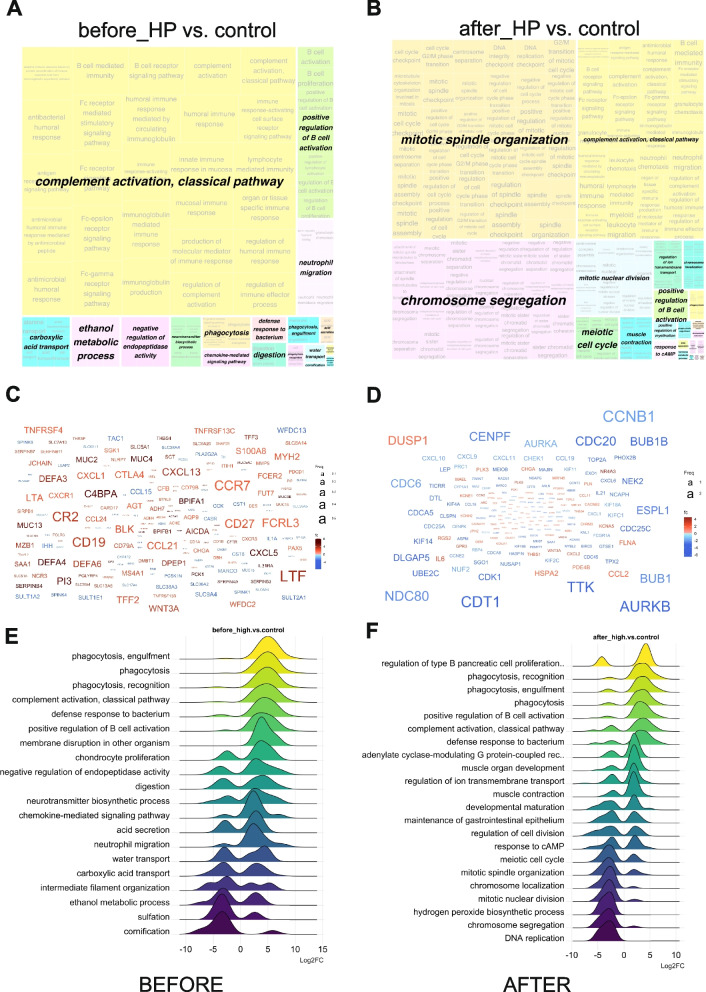
Gene Ontology Over-Representation Analysis of regulated mRNAs in HP group before and after de-obstruction. **A** and **B** treemap views of GO-term clusters (Biological processes, BPs), where each tile and colour represent a term and cluster, respectively. The list of GO terms was converted into a semantic similarity matrix using binary cut method. Tile size and group representatives of each cluster are corresponding to the GO terms’ size. **C** and **D** Word clouds of up-regulated (in red) and down-regulated (in blue) mRNAs, font size corresponding to the frequency of appearance in GO BPs. **E** and **F** Ridge plots of GO ORA showing average Log2FC of the main enriched genes. **A**, **C** and **E** treemap, word cloud and ridge plot for HP group before_HP vs. control DEGs, (B, D and F) treemap, word cloud and ridge plot for HP group after_HP vs. control DEGs

In order to gain an insight into the biological processes in high-pressure BPO before and after de-obstruction, we resorted to Gene Ontology (GO) Over Representation Analysis (ORA). We created a semantic similarity matrix, based on the information content of their most informative common ancestor (Resnik method), for a given list of GO terms. The terms that are closer in the hierarchy or share more common ancestors are likely to be more semantically similar. After removing the redundant GO terms based on a semantic similarity score threshold of 0.7, remaining GO terms were grouped by clustering the semantic similarity matrix using the binary cut method. A treemap view of GO term clusters, where each tile and colour represent a term and cluster respectively, is shown in Fig. 3A for the DEGs in HP group before TURP compared to controls. In the treemap plot, the space used by the cluster is proportional to the number terms in the given cluster. Classical pathway of complement activation was the predominant biological process, with a number of other immune-related processes present (B cell activation, neutrophil migration, phagocytosis). After de-obstruction the overall picture of the regulated biological processes significantly changed, with the cell division-related GO terms becoming predominant (Fig. 3B).

The word clouds in Fig. 3C and D illustrate the hubs of signalling involved in the dysregulated processes, and while most of them are up-regulated in HP bladders before TURP (Fig. 3C), a large proportion becomes down-regulated after TURP (Fig. 3D). Ridge plots indicated a shift in the gene expression: while most immune-related processes were up-regulated in the “before” HP samples (Fig. 3E), after TURP there was a down-regulation of genes involved in DNA replication and cell division and up-regulation of genes related to muscle contractility, while the Log2 fold change (FC) of the genes involved in the immune response processes was reduced but did not revert to the control levels, pointing to ongoing inflammatory processes in the HP bladders 3 months after TURP (Fig. 3F).

### Effects of de-obstruction on dysregulated biological processes in the medium pressure (MP) group

**Fig. 4:**
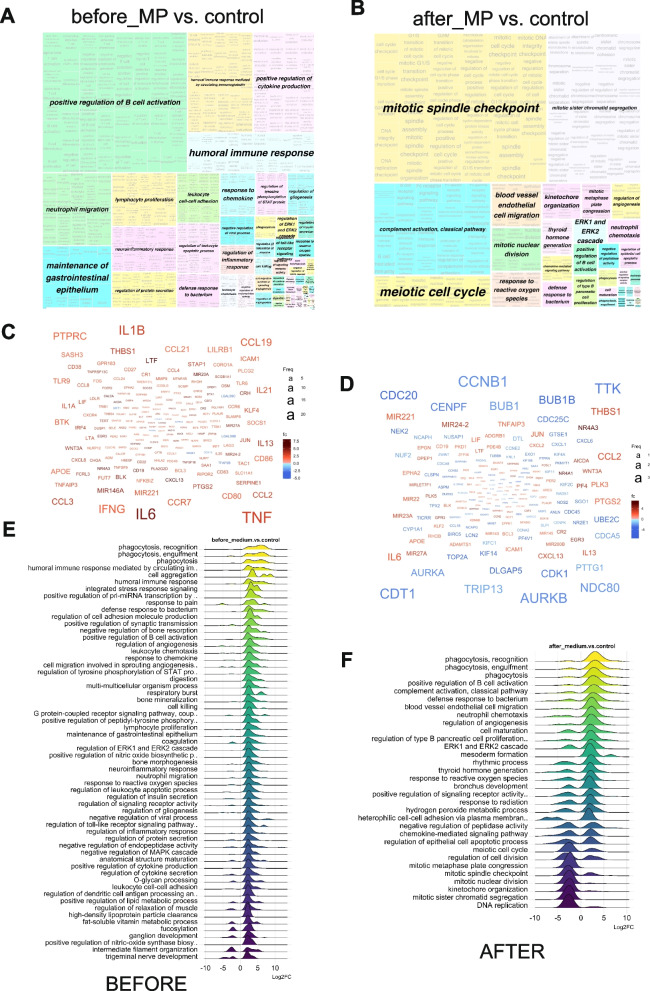
Gene Ontology Over-Representation Analysis of regulated mRNAs in MP group before and after de-obstruction. **A** and **B** treemap views of GO-term clusters (Biological processes, BPs), where each tile and colour represent a term and cluster, respectively. The list of GO terms was converted into a semantic similarity matrix using binary cut method. Tile size and group representatives of each cluster are corresponding to the GO terms’ size. **C** and **D** Word clouds of up-regulated (in red) and down-regulated (in blue) mRNAs, font size corresponding to the frequency of appearance in GO BPs. **E** and **F** Ridge plots of GO ORA showing average Log2FC of the main enriched genes. **A**, **C** and **E** treemap, word cloud and ridge plot for MP group before_MP vs. control DEGs, **B**, **D** and **F** treemap, word cloud and ridge plot for MP group after_MP vs. control DEGs

In accordance with the higher number of DEGs in “before” MP samples (Fig. 2A), there was a considerably higher amount of GO BPs in “before” MP vs. control than in “before” HP-before vs. control (Fig. 4A). Here the predominant processes were immune response, reflected by the highly upregulated hubs including TNF, IL1B and IL6 (Fig. 4C). Similar to the HP group, TURP resulted in significant changes in cell proliferation processes (Fig. 4B) and a general down-regulation of the main signalling molecules in the “after” MP dataset (Fig. 4D). While ridge plots of the MP samples showed up-regulation of the gene expression in the dysregulated pathways before TURP (Fig. 4E), there was a down-regulation of gene sets involved in DNA replication and mitotic cell division after TURP, while the genes responsible for immune response processes remained activated (Fig. 4F).

We analysed the expression of selected genes, corresponding to the defined morphological compartments of the bladder. The detrusor-specific genes showed higher expression levels in the HP group compared to both controls and the MP group (Supplementary Fig. S1A). TURP resulted in up-regulation of the detrusor genes in both HP and MP groups (“after” datasets), indicative of the detrusor changes in line with an improvement of bladder contractility. Urothelial genes, which were significantly down-regulated in the HP group before TURP, have slightly increased but did not reach control levels (Supplementary Fig. S1B). Similarly, TURP did not have pronounced effects on the expression levels of fibroblast genes (Supplementary Fig. S1C).

### Proteomics analysis and integration of transcriptome and proteome data

**Fig. 5:**
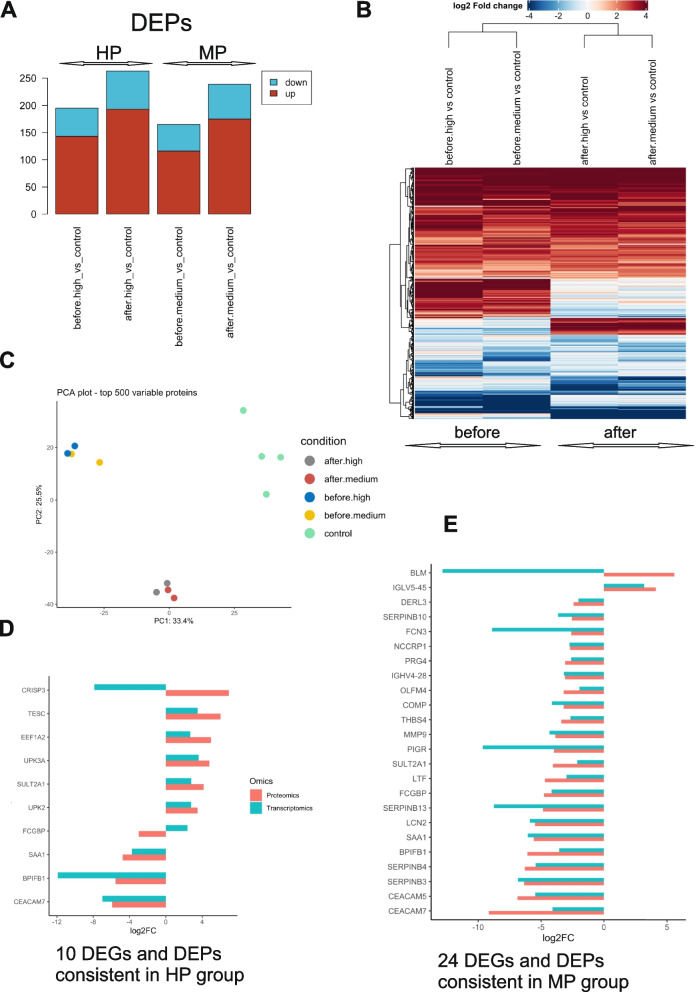
Differentially regulated proteins in BPO groups. **A** Total number of differentially expressed proteins (DEPs) in HP and MP groups compared to control before and after de-obstruction (adjusted *p*-value< 0.1). **B** Heatmap and hierarchical clustering based on Log2FC of normalized expression values of all DEPs compared to controls with *p*-value< 0.05. Proteins are represented in y-axis and patients’ samples before and after de-obstruction are shown in x-axis. One minus Pearson correlation metric was used for clustering accompanied with average linkage method. **C** Principal component analysis using top 500 variable proteins in HP and MP samples before and after TURP, and control samples. **D** Ten differentially expressed mRNAs and proteins in HP group before TURP. **E** 24 differentially expressed mRNAs and proteins in MP group before TURP. Regulation of protein levels (proteomics) is shown in orange, and mRNA (transcriptomics) shown in blue

The proteins were extracted from the bladder biopsies and analysed by nanoLC-MS/MS as described in Methods. The number of differentially expressed proteins (DEPs) in each group was similar, with an average of 143 up- and 52 down-regulated DEPs in before_high, 193 up- and 70 down regulated DEGs in after_high, 116 up- and 49 down-regulated in before_medium and 174 up- and 64 down-regulated DEPs after_medium compared to controls (Fig. 5A).

Hierarchical clustering analysis revealed a high proteome similarity between the HP and MP groups before TURP (Fig. 5B). There was a shift in the protein composition but preserved inter-group similarity after TURP (Fig. 5B), which was confirmed by PCA using top 500 variable proteins: before_high clustered together with before_medium and away from the after_high/after_medium cluster. Both clusters are well separated from the controls, indicating significant differences in the proteomes of BPO, which were not completely normalized by TURP (Fig. 5C). In the HP group, 10 DEPs were shared with DEGs, with 8 proteins showing similar regulation (Fig. 5D) In the MP group 24 DEGs and DEPs were consistent, with only one being oppositely regulated (Fig. 5E).

**Fig. 6:**
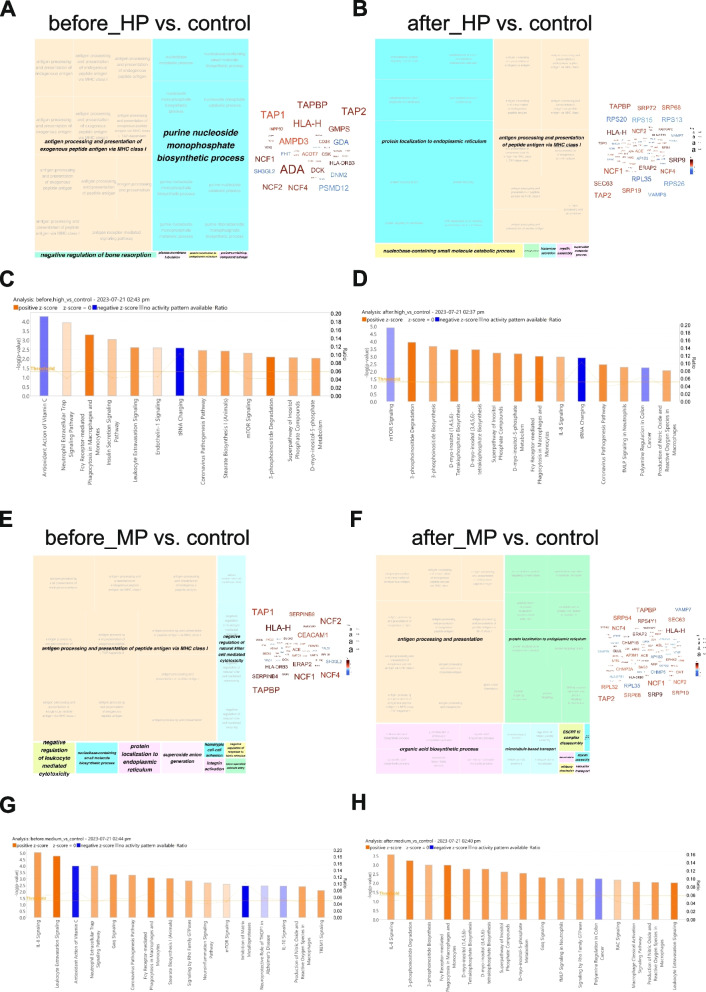
Gene Ontology Over-Representation Analysis and IPA pathway analysis of regulated proteins in HP and MP BPO groups before and after de-obstruction Treemap views of GO-term clusters (Biological processes, BPs) for DEPs in HP group before (**A**) and after (**B**) deobstruction, compared to controls. Word clouds of up-regulated (in red) and down-regulated (in blue) proteins, font size corresponding to the frequency of appearance in GO BPs. Top canonical IPA pathways with non-zero z-score for HP before TURP samples (**C**) and HP after TURP samples (**D**) compared to controls. Treemap views of GO-term clusters (Biological processes, BPs) for DEPs in MP group before (**E**) and after (**F**) de-obstruction, compared to controls. Word clouds of up-regulated (in red) and down-regulated (in blue) proteins, font size corresponding to the frequency of appearance in GO BPs. Top canonical IPA pathways with non-zero z-scores for MP before TURP samples (**G**) and MP after TURP samples (**H**) compared to controls

We carried out GO ORA with DEPs in both sample groups, and in agreement with PCA sample clustering, observed a high degree of similarity: both groups reported antigen processing and presentation as predominant BP before TURP, and the word clouds representing the main hubs of signalling were also similar between the groups (Fig. 6A, E).

Neutrophil Cytosolic Factor proteins NCF, and transporter associated with antigen processing (TAP) were prominent in both datasets, consistent with antigen processing and presentation processes being activated (Fig. 6A, E). IPA showed similarities in the activated pathways, however, the MP group had a higher number of immune-related pathways (Fig. 6C and G). De-obstruction resulted in partial restoration of altered BPs in both groups (Fig. 6B for HP, Fig. 6F for MP), however, IL8 signalling remained the main activated pathway in the MP group (Fig. 6G and H). mTOR signalling, which was activated in the HP group before TURP, was inhibited after TURP, in line with reduced metabolic and growth activity (Fig. 6C and D). 3-phosphoinositide biosynthesis and degradation pathways became prominent in both groups after TURP (Fig. 6D and H). In line with the transcriptome analysis of the expression levels of urothelial genes (Fig. S1B) which showed strong down-regulation of UPK1A, UPK2 and UPK3A genes in particular in the “before” HP mRNA dataset, we observed reduced levels of UPK2 and UPK3A proteins in the HP group before TURP (before_high, Supplementary Fig. S2A).

### De-obstruction normalized SOX21 expression levels and affected their potential target genes

**Fig. 7:**
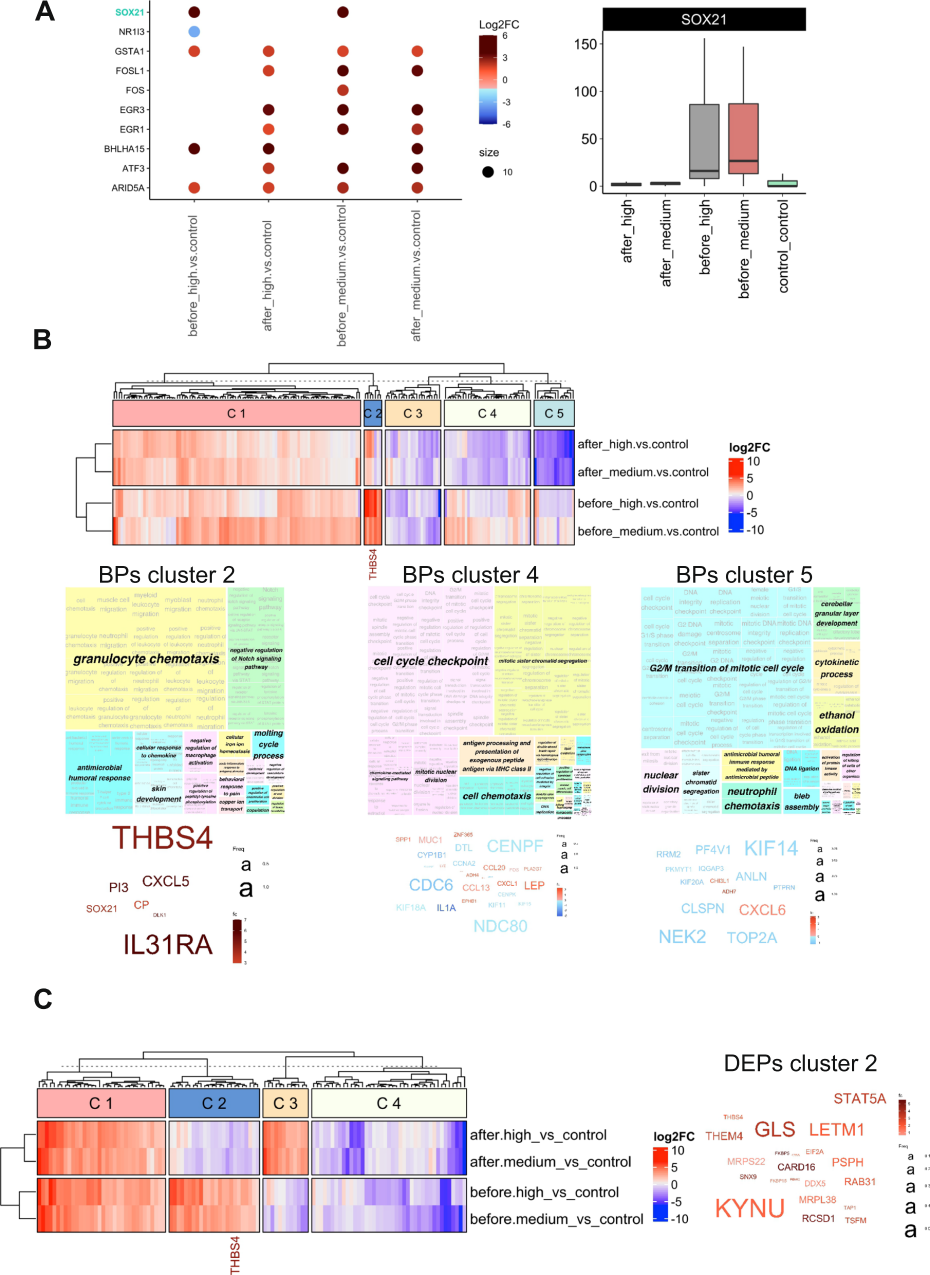
Transcription factors (TFs) regulated by BPO and role of SOX21 in obstruction-driven bladder remodelling. **A** Bubble plot of TFs detected regulated in BPO datasets. Log2FC of up-regulated (in brown-red) and down-regulated (in blue) TF are shown in each dataset (HP before and after TURP, MP before and after TURP) compared to controls. A graph shows normalized read counts for SOX21 mRNA in all samples. **B** DEGs regulated by SOX21 and their involvement in biological processes. Heatmap and hierarchical clustering of all predicted SOX21 mRNA targets in BPO datasets based on Log2FC. Treemap views of GO-term clusters (Biological processes, BPs) for DEGs in clusters 2, 4 and 5, which showed regulation after de-obstruction. Word clouds of up-regulated (in red) and down-regulated (in blue) DEGs in each cluster, font size corresponding to the frequency of appearance in GO BPs, regulation based on log2FC values in before_high vs. control transcriptome dataset. **C** DEPs regulated by SOX21 and their involvement in biological processes. Heatmap and hierarchical clustering of all predicted SOX21 protein targets in BPO datasets based on Log2FC. Treemap views of GO-terms (Biological processes, BPs) for DEGs in cluster 2, which showed regulation after de-obstruction. Word clouds of up-regulated (in red) and down-regulated (in blue) DEPs in cluster 2, font size corresponding to the frequency of appearance in GO BPs, regulation based on log2FC values in before_high vs. control proteome dataset

In order to gain insights into the regulation of transcription factors (TFs) during BPO and de-obstruction, we mapped the DEGs identified in the “before” HP and “before” MP datasets against the available list of TFs from TRED [23], ITFP [25], TRRUST [22], and Marbach [24] databases. Ten TFs, expressed and regulated in the BPO samples are shown in Fig. 7A. Most TFs were up-regulated in BPO before TURP and persisted at a high level 3 months after TURP: ARID5A, GSTA1, EGR1, EGR3, ATF3, FOSL1. In contrast, SOX21 was up-regulated in both BPO groups HP and MP, but normalized after TURP (Fig. 7A). We further investigated its relevance for gene expression regulation by mapping all its potential target mRNAs in all four datasets compared to control (before_high, before_medium, after_high and after_medium) (Fig. 7B). The mRNAs, predicted to be regulated by SOX21, cluster in 5 groups, with clusters C2, C4 and C5 showing the expected down-regulation in the “after” datasets (the heatmap in Fig. 7B), when SOX21 itself has returned to the normal levels (Fig. 7A). Using the target mRNA levels from clusters 2, 4 and 5 “before” HP dataset as a source, we investigated the regulated BPs and most significant signalling hubs in each cluster (Fig. 5B). BPs in cluster 2 are mostly immune response, with thrombospondin THBS4 and interleukin 31 receptor IL31RA being the most important pathway components. BPs in clusters 4 and 5 are responsible for cell cycle regulation, and after SOX21 mRNA levels returned to control after TURP, the genes involved in these pathways were down-regulated. The transcriptome data on SOX21 target expression is in agreement with the proteome results in cluster C2 (Fig. 7C) – most proteins in this cluster were up-regulated before TURP, when SOX21 mRNA levels were high, and returned to control levels or were down-regulated after TURP. THBS4 is a component of this DEPs cluster, controlling some of the BPs (word cloud, Fig. 7C). The mRNA and protein levels of SOX21 target THBS4 were increased in HP and MP groups before TURP, and returned to control levels after TURP (Supplementary Fig. S2B).

## Discussion

BPO induces significant remodelling in the human urinary bladder, which demonstrates a very similar reaction to increased outlet resistance as the heart subjected to pressure overload [34]. Based on the observations made in animal models of pBOO, BOO is a chronic gradually progressive disease. Hypertrophy is the bladder’s initial response which most likely advances further until the final decompensatory stage, loss of contractility [35]. On the molecular level, these processes are characterised by hypoxia and inflammatory response, which induce organ fibrosis [5]. Indirect evidence collected in humans also points to the fact that BPO is a progressive disease, which can be delayed by pharmacological treatment with alpha-1 adrenergic antagonists and/or 5-alpha reductase inhibitors [36, 37]. In humans, monitoring urodynamic changes in the obstructed bladder after TURP allows the assessment of functional recovery, although drawing conclusions about the morphological alterations in the affected organ is difficult because a very limited number of studies offer relevant follow-up information. Increased bladder pressure and a reduced flow rate are the physiological parameters seen to be improved by therapeutic measures including surgical de-obstruction. Ultrasound measurements of the bladder or detrusor wall thickness, indicative of muscle hypertrophy, have been proposed to non-invasively monitor bladder remodelling during BPO, with significant differences observed between obstructed and non-obstructed patients [38, 39]. Bladder wall thickness was decreased one month after TURP surgery, indicating a recovery trend after de-obstruction [40]. However, there were no symptomatic or urodynamic gains from de-obstruction in men with BPO and detrusor underactivity [41], implying that the timing of surgery is crucial for the outcome. Indeed, our previous study of the molecular changes in bladder dome biopsies from patients with different urodynamic phenotypes [6] showed profound gene expression changes in BPO-induced detrusor underactivity leading to the loss of contractility.

This study investigated the changes in cell signalling processes within BPO-affected bladders before and after de-obstruction. The analysis focused on examining the transcriptomes and proteomes of bladder dome biopsies collected from men who experienced urodynamically confirmed functional improvement following TURP. Age-matched patients with BPO without DO were divided into two groups based on the PdetQmax values recorded by UDI before de-obstruction: high and medium pressure (HP and MP) groups. PdetQmax was the only statistically significant parameter, separating the HP and MP groups (Fig. 1), however, the MP group had a slightly higher residual volume (RV) and a considerably lower BCI, although the differenece in BCI did not reach statistical significance. Three months after de-obstruction surgery, the voiding parameters PdetQmax, Qmax and RV were significantly improved in both groups, without any significant inter-group difference in the values after TURP.

The small number of patients per group (*n* = 3) was a limitation of this study, which did not allow certain observed trends in the UDI parameters to reach statistical significance. The mean age of the control patients (*n* = 6) was lower than in patients with BPO, because due to the increasing age-related prevalence of BPO in the male population, it was impossible to recruit truly age-matched controls without any LUTS.

The overall number of gene expression changes in both groups compared to controls without LUTS showed an increased number of DEGs in the MP group before TURP, and comprehensive bioinformatics analysis revealed 10 molecular classifiers (CYP1B1, TIPARP, AREG, FOXE1, CYSRT1, PLAAT2, OVOL1, MYBPC1, CDX2, CYP1A1) reliably differentiating between the HP and MP groups before TURP in PCA. The proteins encoded by these genes contribute to oxidative homeostasis (CYP1B1, CYP1A1, CYSRT1), are transcription factors (CDX2, FOXE1 which targets TGF-beta, EGF/TGF-family member AREG, OVOL1), are involved in muscle contraction (MYBPC1) and immune function (TIPARP). Comparison of bladder transcriptomes before and after TURP in the HP group revealed 12 markers (CXCL13, BHMT, EGR3, CCL19, CCL21, NR4A3, CRTAC1, SAA1, UPK2, NR4A1, CCL18, UPK1A), discriminating the samples collected at two time-points from the same patients, indicating that de-obstruction induced significant alterations in the gene expression profiles of the affected bladders. Interestingly, here in addition to transcription factors (NR4A1, BHMT, EGR3, NR4A3) and inflammatory markers (CXCL13, CCL19, CCL21, CCL18 and SAA1) we discovered two uroplakin genes (UPK2 and UPK1A). Further analysis revealed that these genes were significantly down-regulated before TURP in the HP group, compared to controls and the MP group. Their expression was improved by de-obstruction but did not reach control levels. A combination of the 22 markers, used in PCA, revealed that before TURP the MP group was highly different from both the HP group and the controls. Although de-obstruction did not completely restore gene expression in the HP and MP groups, the resulting profiles in the “after” samples were more similar to each other and closer to the controls. Thus, our unbiased bioinformatics analysis of the whole transcriptomes revealed a partial normalization of gene expression, in line with functional improvement observed by UDI.

The analysis of the biological processes and activated pathways in HP and MP groups before and after TURP showed considerable improvement but no compete reversal of the BPO-induced bladder gene expression deterioration. Activation of the immune response processes was a prominent feature in both patient groups before TURP, but the hallmarks of inflammation and the main activated pathways were strikingly different in both groups before TURP. Complement activation was the main up-regulated process in the HP group, whereas the MP group showed more advanced signs of immune response, including significant up-regulation of TNF-driven inflammation, and concomitant IL6, IL1B and PTGS2 up-regulation. B cell activation, neutrophil migration and humoral immune response with activated cytokine production were the top BPs in MP “before” samples, while the complement activation was the top BP in HP “before” samples. These differences, together with the higher number of DEGs, might be an indication of a progressive bladder deterioration in the MP group in response to BPO. Complement activation is becoming increasingly recognized as a key contributor to the beginning sterile inflammation, when the damaged tissues release danger signals and trigger complement, which acts on a range of leukocytes to augment and bridge the innate and adaptive immune systems [42]. Complement triggers phagocytosis [43] and the subsequent neutrophil infiltration, observed in the MP group. Thus, the changes in immune response in the obstructed bladder might serve as an indicator of the disease progression. Likewise, our earlier study [6] showed a steady increase of DEGs in obstructed acontractile patients (UA group) compared to those who were still able to void (BO group). After TURP there was a compensatory down-regulation of many affected processes in both groups, particularly those controlling cell division and cell cycle progression. Interestingly, the expression levels of detrusor muscle genes, which were already significantly up-regulated in the HP “before” samples but down-regulated in MP “before” samples, were elevated following TURP in both groups, accompanied by the activation of muscle- and contractility-related pathways. This indicates that de-obstruction was beneficial for bladder contractility. In the “before” MP group, we observed activated TNF-driven signalling and concomitant down-regulation of detrusor gene expression. This could be an indication of the adverse effects of bladder inflammation on smooth muscle contractility, as previously described in TNF-alpha treated SMCs in vitro [44], and as a consequence result in the lower PdetQmax before TURP compared to the HP group, where no such processes were recorded. The down-regulated BPs of “cornification” and “intermediate filament organization” in the HP group “before” samples contain many urothelial genes, including uroplakins, all of which were significantly down-regulated. This might be indicative of urothelial dysfunction, exacerbated by high bladder pressure, in humans similar to the animal models of pBOO [45, 46]. A previous study showed significantly lower expression of E-cadherin, and a higher number of apoptotic cells in humans with BPO [47], confirming the adverse effects of BOO on urothelial morphology and function.

Proteome analysis indicated a significant difference in protein composition between the ‘before’ and ‘after’ TURP states in both the HP and MP groups. Overall, less DEPs were detected (195 for HP, 165 for MP before TURP compared to controls) compared to the DEGs (855 for HP, 1496 for MP before TURP compared to controls). Immune processes were highly regulated in the proteomes of BPO patients and showed partial normalization after de-obstruction. We also observed changes in the metabolic and proliferative processes, evident by alteration of mTOR and 3-phosphoinositide biosynthesis and degradation pathways after TURP.

To comprehend the impact of obstruction on the factors driving gene expression changes, we investigated the expression levels of known or predicted transcription factors (TFs) and regulators in the transcriptomes of all bladder biopsies before and after TURP. In particular, we looked for TFs which were altered in the “before” state and normalized 3 months after surgery. Only 2 TFs matched these criteria: SOX21, which was significantly up-regulated in both HP and MP groups, and NR1I3 which was specifically down-regulated in the HP group.

The SRY-Box Transcription Factor 21 (SOX21) participates in regulating cell proliferation and differentiation across various tissues [48]. A database search was performed to identify known and predicted mRNA targets regulated by SOX21. Subsequently, using the mRNA levels of these targets we performed hierarchical clustering analysis to examine the correlation of their expression level changes with the up-regulation of SOX21 and its subsequent normalization. We identified three distinct gene clusters, denoted as Cluster 2, Cluster 4, and Cluster 5, consisting of 7, 34, and 16 genes, respectively, which were regulated in accordance with SOX21 levels before and after TURP. Genes in Cluster 2 were involved in the immune-related BPs (granulocyte chemotaxis, antimicrobial response, etc.) with thrombospondin THBS4 being a prominent signalling molecule; these genes were highly elevated in “before” HP and MP and reduced after TURP. The genes in Clusters 4 and 5 were related to the processes of cell division and chemotaxis; they were significantly elevated in the MP group, and down-regulated after de-obstruction. The proteome analysis of SOX21 targets revealed one protein cluster, also containing THBS4 and significantly down-regulated concomitant with normalization of SOX21 expression.

THBS4 is a glycoprotein mediating cell-to-cell and cell-to-matrix interactions. It is involved in cellular proliferation, migration, adhesion and attachment, inflammatory response and adaptive responses of the heart to pressure overload and in myocardial function and remodelling [49]. THBS4 was induced after outlet obstruction in rodents, and considered to be a sensitive marker of obstruction, although its knock-out in mice did not affect bladder growth or repression of contractile markers [50]. Here we confirm the up-regulation of THBS4 in human BPO at both protein and mRNA levels, and its normalization 3 months after de-obstruction. Importantly, THBS4 can be detected in urine as was shown in human urinary proteomics studies from healthy and diseased individuals [51], making it a potential non-invasive BPO biomarker candidate, if its levels in the urine from men with BPO correlate with the functional impairment caused by obstruction.

Here we established a possible link between SOX21 up-regulation in obstructed bladders, and an increased cell proliferation leading to organ hypertrophy. Earlier studies showed that SOX21 suppressed differentiation of airway progenitor cells during lung development and promoted cell division [52]. Similarly, higher concentration of SOX21 inhibited neuron formation and instead promoted progenitor maintenance [48]. These data are in agreement with our observation that SOX21 targets in the Clusters 4 and 5 regulate cell cycling and proliferation pathways. Elucidating the mechanisms, which induce the high responsiveness of SOX21 to obstruction would be important for understanding the progressive changes caused by BPO and may represent a novel approach for the diagnosis and treatment of BPO.

## Conclusions

We present the first comprehensive characterization of the bladder gene expression changes in the human patients with BPO before and 3 months after TURP. We demonstrated that the transcriptome profiles and predicted biological processes were different in the bladders with significantly different PdetQmax before de-obstruction, and the patients with average PdetQmax 55 ± 21.7 cmH2O before TURP and lower BOOI and BCI showed a more advanced organ deterioration compared to the patients with 107 ± 20.4 cmH2O before TURP and higher BOOI. Our findings reveal substantial yet incomplete normalization of cell signalling pathways three months after TURP, consistent with improved urodynamic parameters. Transcription factor SOX21 and its target mRNAs including thrombospondin THBS4, regulating pivotal immune and proliferation processes, were highly sensitive to de-obstruction. Our study suggests that a combination of UDI, and a preferably non-invasive indicator of the immune response activation in the bladder might be beneficial when selecting an optimal intervention point to mitigate loss of contractility.

### Supplementary Information

**Additional file 1: Supplementary Figure S1.** Gene groups regulated during de-obstruction in HP and MP bladders. **Supplementary Figure S2.** Expression of uroplakin genes and THBS4 and their regulation after de-obstruction. transcriptomics_DEGs. proteomics_DEPs. 22gene signature. Sox21_ClusterC2_DEPs. Sox21_ClusterC2_DEGs. Sox21_ClusterC4_DEGs. Sox21_ClusterC5_DEGs. session_info.

## Data Availability

The mRNA datasets generated and analysed during the current study are available in the European Nucleotide Archive (ENA) repository under ENA accession numbers: Accession PRJEB65173, Secondary Accession ERP150319, the project “Transcriptome Analysis of Bladder Samples Reveals Changes in BOO Patients Pre and Post TURP Surgery” (https://www.ebi.ac.uk/ena/browser/view/PRJEB65173). The mass spectrometry proteomics data have been deposited to the ProteomeXchange Consortium via the PRIDE [53] partner repository with the dataset identifier PXD044777. The proteomics data are available from the authors upon reasonable request, and freely available after the publication date. The lists of genes used for downstream analysis and regulated genes involved in signalling pathways are available as supplementary files.

## Abbreviations

BPO: Benign prostatic obstruction
NGS: Next-generation sequencing
DEGs: differentially expressed genes
DEPs: differentially expressed proteins
mRNA: messenger RNA
GO ORA: Gene ontology over-representation analysis
QPCR: quantitative real-time polymerase chain reaction
FC: fold change
n.s.: not significant
