# Supplementary material for: De-obstruction of bladder outlet in humans reverses organ remodelling by normalizing the expression of key transcription factors
Source: BMC Urol. 2024 Feb 7;24:33. doi: 10.1186/s12894-024-01417-8 (PMC10848355; doi:10.1186/s12894-024-01417-8)
Supplement: Supplementary file 1 — Additional file 1: Supplementary Figure S1. Gene groups regulated during de-obstruction in HP and MP bladders. Supplementary Figure S2. Expression of uroplakin genes and THBS4 and their regulation after de-obstruction. transcriptomics_DEGs. proteomics_DEPs. 22gene signature. Sox21_ClusterC2_DEPs. Sox21_ClusterC2_DEGs. Sox21_ClusterC4_DEGs. Sox21_ClusterC5_DEGs. session_info. [file 12894_2024_1417_MOESM1_ESM.zip › Supplementary Figures with Legends.pdf]

Supplementary Figure Legends

Supplementary Figure S1. Gene groups regulated during de-obstruction in HP and MP bladders.

Bubble plots of selected regulated mRNAs in BPO datasets. Log2FC of up-regulated (in brown-red) and down-regulated (in blue) genes are shown in each dataset (HP before and after TURP, MP before and after TURP) compared to controls. Individual graphs for each mRNA show normalized read counts in each sample group.

(A) Detrusor genes, (B) urothelium genes and (C) fibroblast genes.

Supplementary Figure S2. Expression of uroplakin genes and THBS4 and their regulation after de-obstruction

(A) mRNA and protein levels of detected uroplakin mRNAs and proteins in sample groups and controls. Shown are normalized read counts (mRNA) and intensities (proteomics).

(B) mRNA and protein levels of THBS4 in all sample groups and controls.

Supplementary files

transcriptomics_DEGs.xlsx

proteomics_DEPs.xlsx

22gene signature.xlsx

Sox21_ClusterC2_DEPs.xlsx

Sox21_ClusterC2_DEGs.xlsx

Sox21_ClusterC4_DEGs.xlsx

Sox21_ClusterC5_DEGs.xlsx

session_info.csv

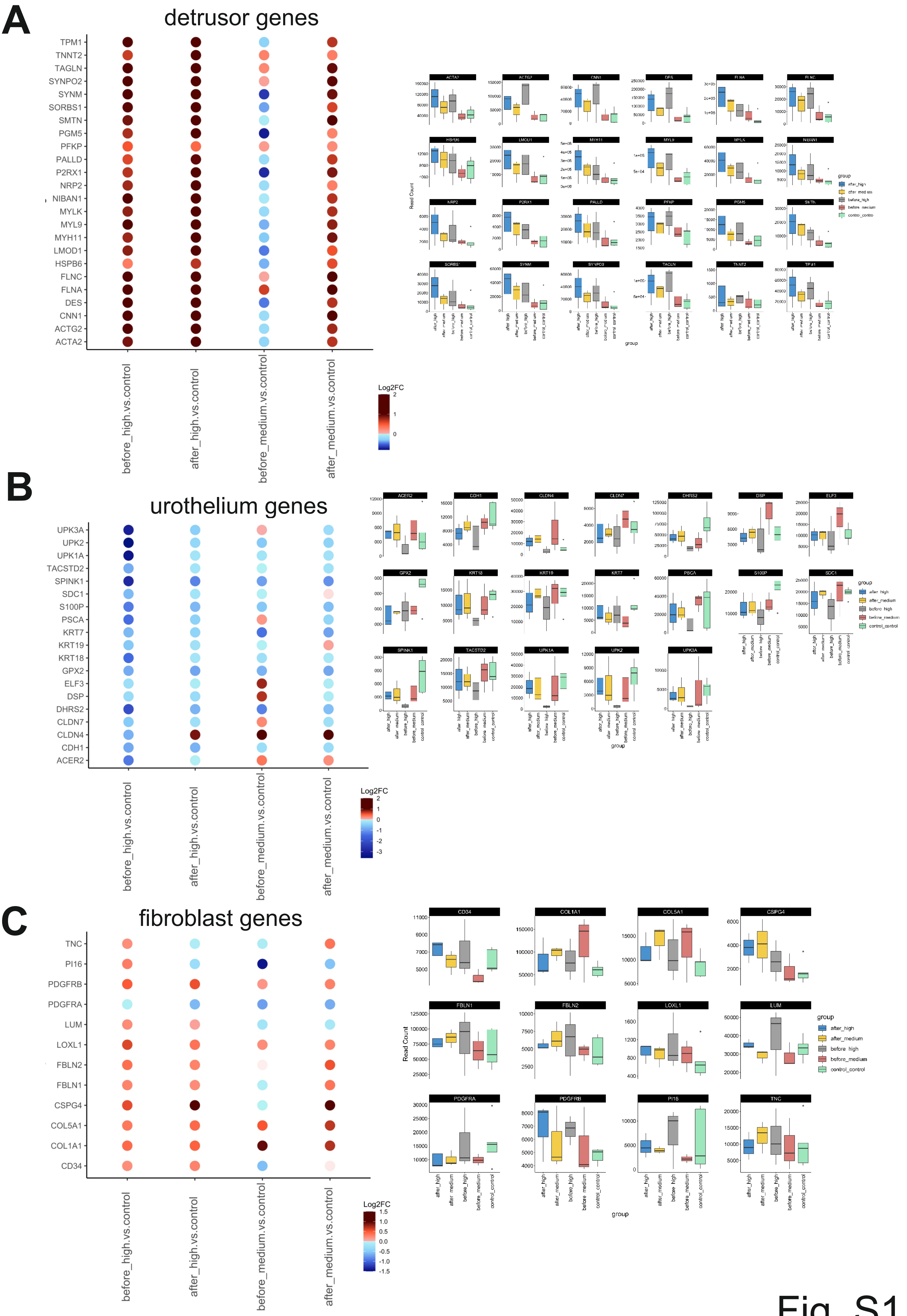

A
detrusor genes
TPM1
TNNT2
TAGLN
SYNPO2
SYNM
SORBS1
SMTN
PGM5
PFKP
PALLD
P2RX1
NRP2
NIBAN1
MYLK
MYL9
MYH11
LMOD1
HSPB6
FLNC
FLNA
DES
CNN1
ACTG2
ACTA2
before_high.vs.control
after_high.vs.control
before_medium.vs.control
after_medium.vs.control
Log2FC
Read Count
group
B
urothelium genes
UPK3A
UPK2
UPK1A
TACSTD2
SPINK1
SDC1
S100P
PSCA
KRT7
KRT19
KRT18
GPX2
ELF3
DSP
DHRS2
CLDN7
CLDN4
CDH1
ACER2
before_high.vs.control
after_high.vs.control
before_medium.vs.control
after_medium.vs.control
Log2FC
group
after_high
after_medium
before_high
before_medium
control_control
C
fibroblast genes
TNC
PI16
PDGFRB
PDGFRA
LUM
LOXL1
FBLN2
FBLN1
CSPG4
COL5A1
COL1A1
CD34
before_high.vs.control
after_high.vs.control
before_medium.vs.control
after_medium.vs.control
Log2FC
Read Count
group
after_high
after_medium
before_high
before_medium
control_control

Fig. S1

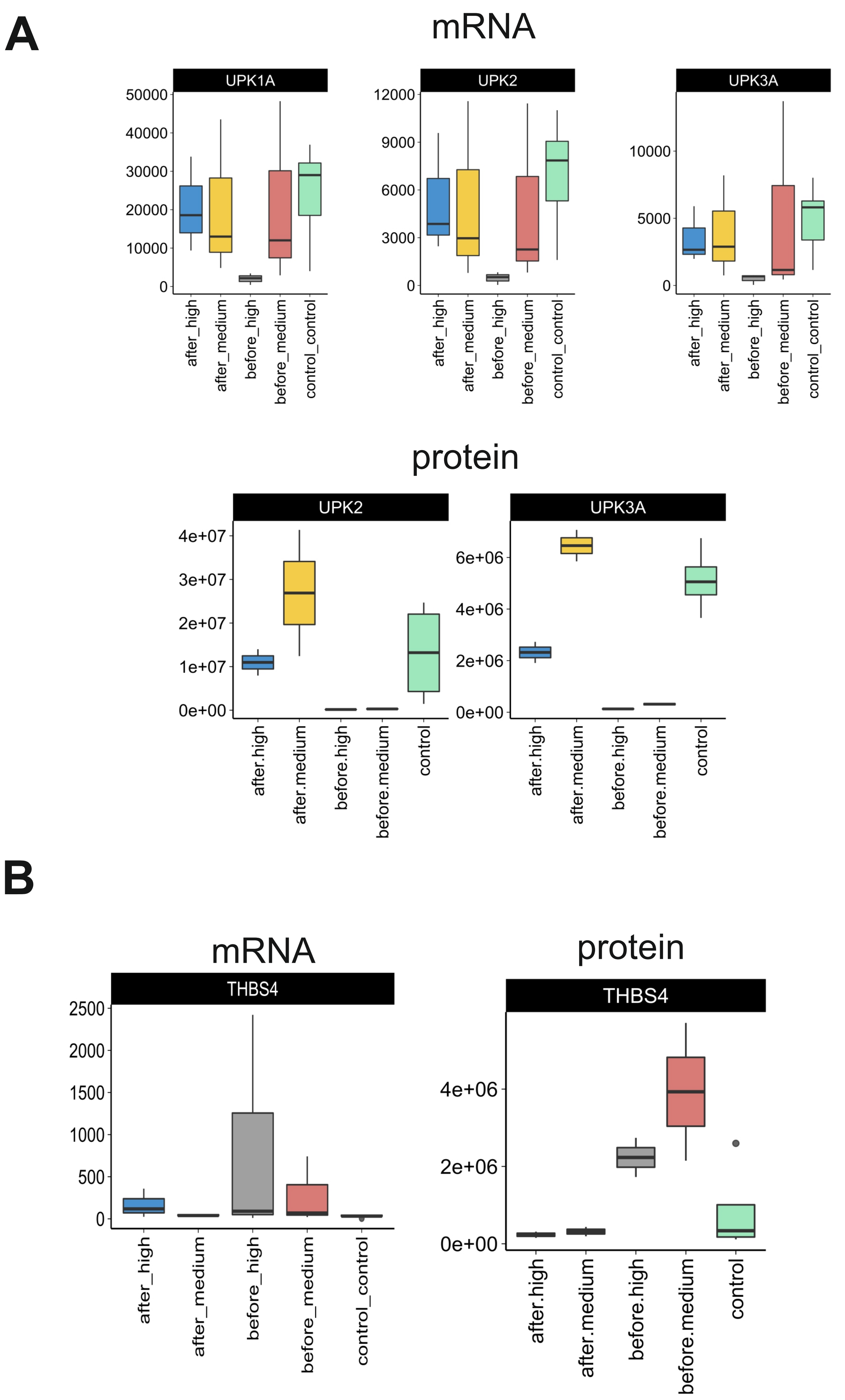
A
mRNA
UPK1A
UPK2
UPK3A
50000
40000
30000
20000
10000
0
12000
9000
6000
3000
10000
5000
after_high
after_medium
before_high
before_medium
control_control
protein
UPK2
UPK3A
4e+07
3e+07
2e+07
1e+07
0e+00
6e+06
4e+06
2e+06
after.high
after.medium
before.high
before.medium
control
B
mRNA
THBS4
2500
2000
1500
1000
500
protein
THBS4
4e+06
2e+06
0e+00

Fig. S2
